# Photosynthetic Light Responses May Explain Vertical Distribution of Hymenophyllaceae Species in a Temperate Rainforest of Southern Chile

**DOI:** 10.1371/journal.pone.0145475

**Published:** 2015-12-23

**Authors:** María José Parra, Karina I. Acuña, Angela Sierra-Almeida, Camila Sanfuentes, Alfredo Saldaña, Luis J. Corcuera, León A. Bravo

**Affiliations:** 1 Departamento de Ciencias Biológicas y Químicas, Facultad de Ciencia, Universidad San Sebastián, Cruz 1577, Concepción, Chile; 2 Departamento de Botánica, Facultad de Ciencias Naturales y Oceanográficas, Universidad de Concepción, Casilla 160-C, Concepción, Chile; 3 Instituto de Ecología y Biodiversidad (IEB), Casilla 653, Santiago, Chile; 4 Departamento de Ciencias Ecológicas, Facultad de Ciencias, Universidad de Chile, Las Palmeras 3425, Chile; 5 Laboratorio de Fisiología y Biología Molecular Vegetal, Departamento de Ciencias Agronómicas y Recursos Naturales. Facultad de Ciencias Agropecuarias y Forestales & Center of Plant, Soil Interactions and Natural Resources Biotechnology, Scientific and Technological Bioresource Nucleus, Universidad de La Frontera, Casilla 54-D, Temuco, Chile; Estacion Experimental de Zonas Aridas - CSIC, SPAIN

## Abstract

Some epiphytic Hymenophyllaceae are restricted to lower parts of the host (<60 cm; 10–100 μmol photons m^-2^ s^-1^) in a secondary forest of Southern Chile; other species occupy the whole host height (≥10 m; max PPFD >1000 μmol photons m^-2^ s^-1^). Our aim was to study the photosynthetic light responses of two Hymenophyllaceae species in relation to their contrasting distribution. We determined light tolerance of *Hymenoglossum cruentum* and *Hymenophyllum dentatum* by measuring gas exchange, PSI and PSII light energy partitioning, NPQ components, and pigment contents. *H*. *dentatum* showed lower maximum photosynthesis rates (A_max_) than *H*. *cruentum*, but the former species kept its net rates (A_n_) near A_max_ across a wide light range. In contrast, in the latter one, A_n_ declined at PPFDs >60 μmol photons m^-2^ s^-1^. *H*. *cruentum*, the shadiest plant, showed higher chlorophyll contents than *H*. *dentatum*. Differences in energy partitioning at PSI and PSII were consistent with gas exchange results. *H*. *dentatum* exhibited a higher light compensation point of the partitioning of absorbed energy between photochemical Y(PSII) and non-photochemical Y(NPQ) processes. Hence, both species allocated energy mainly toward photochemistry instead of heat dissipation at their light saturation points. Above saturation, *H*. *cruentum* had higher heat dissipation than *H*. *dentatum*. PSI yield (YPSI) remained higher in *H*. *dentatum* than *H*. *cruentum* in a wider light range. In both species, the main cause of heat dissipation at PSI was a donor side limitation. An early dynamic photo-inhibition of PSII may have caused an over reduction of the Qa^+^ pool decreasing the efficiency of electron donation to PSI. In *H*. *dentatum*, a slight increase in heat dissipation due to acceptor side limitation of PSI was observed above 300 μmol photons m^-2^s^-1^. Differences in photosynthetic responses to light suggest that light tolerance and species plasticity could explain their contrasting vertical distribution.

## Introduction

Although light energy is an essential resource for photosynthesis, both extreme low and high light intensity can limit plant performance [1]. Thus, sunlight is a key factor in determining plants distribution, since it is strictly related to the ability of such organisms to deal with its absorption, photochemical conversion, and harmless dissipation. When light energy is absorbed by chlorophyll molecules, these pigments reach a singlet excited state. To relax back to its ground form, the energy captured by them can basically have one of three fates: (1) it can be re-emitted as fluorescence; (2) it can be used to drive photochemical processes (e.g. photosynthesis, photorespiration, water-water cycle, etc.), or (3) it can be dissipated non-photochemically as heat [2]. These three processes have a competitive kinetics, in such a way that any increase in the efficiency of one will result in a decrease in the yield of the other two. Hence, changes in PSII chlorophyll fluorescence as well as in PSI absorbance provide important information about photochemical and non-photochemical efficiencies for energy dissipation [3–6]. For instance, heat dissipation at PSII level indicates photoprotective processes and/or photoinhibition according to its relaxing kinetic (i.e. qE: fast heat dissipation by xanthophyll cycle, or qI: sustained photoinhibition) [7, 8]. In analogy, heat dissipation by PSI may indicate a donor side limitation due to a shortage of electrons from the intersystem chain to reduce PSI or acceptor side limitation caused for a lack of electrons acceptors that restrain charge separation at PSI level [9]. Nevertheless, if harmless mechanisms of photoprotection (i.e. such as non-photochemical quenching) are insufficient to deal with an excess of absorbed energy and the prevention of photoinhibition, this excess will conduct to damaging free radicals formation (e.g. superoxide anion, hydrogen peroxide, hydroxyl radical, peroxyl radical and singlet oxygen), and to the subsequent photo-oxidative destruction of the photosynthetic apparatus [10, 11].

In natural environments, incident light varies over several orders of magnitude and on a broad time scale that can range from seasons to seconds. Thus, plants have developed a wide spectrum of biochemical and physiological responses to light that enable them to adjust their photosynthetic performance, and consequently, their net carbon gain and plant growth to their light environments [8, 10, 12, 13]. In this context, plants that have a greater capacity to avoid, use and/or dissipate the absorbed energy are frequently found in sunny habitats and they are known as sun plants. In contrast, plants that do not have the same ability are confined to shaded habitats and they are called shade plants [10, 14–16]. Shade plants are characterized by higher CO_2_ assimilation rates at low irradiances, lower light compensation and saturation points, low capacities for photoprotective pathways such thermal energy dissipation, lower chlorophyll *a/b* ratios and higher chlorophyll contents [10, 15]. The Hymenophyllaceae Link. is one of the most attractive, largest, and specious families of basal ferns that include more than 600 species. It exhibits a remarkable diversity in terms of plant morphology and habitat requirements [17]. The most conspicuous features of these plants are a mono or few cell layer fronds (to which they own the alternative name of “filmy ferns” or “filmies”), a highly reduced or absent cuticles, and the complete lack of differentiated epidermis and stomata [18]. These features suggest that filmy ferns have a limited control of gas exchange (i.e. CO_2_, O_2_ and water), and therefore, a restricted distribution. Although Hymenophyllaceae species are globally distributed, they are one of the main components of forests in tropical and temperate-humid regions, being recognized as an important indicator of these ecosystems conservation conditions [17–19]. These ferns diversity and abundance have been observed in the middle and lower strata of the forest, where pteridophytes are adapted to such environmental conditions [20–22]. From an ecophysiological point of view, filmy ferns are generally perceived as shade plants, inhabiting deep shade and constant humid places in the forest [23–26]. However, the latest studies carried out in tropical and temperate environments have reported that some filmy ferns are also able to inhabit the canopy, the top forest strata [22, 27, 28]. This suggests that some of these ferns are able to withstand high light intensities during significant periods of time.

**Fig 1:**
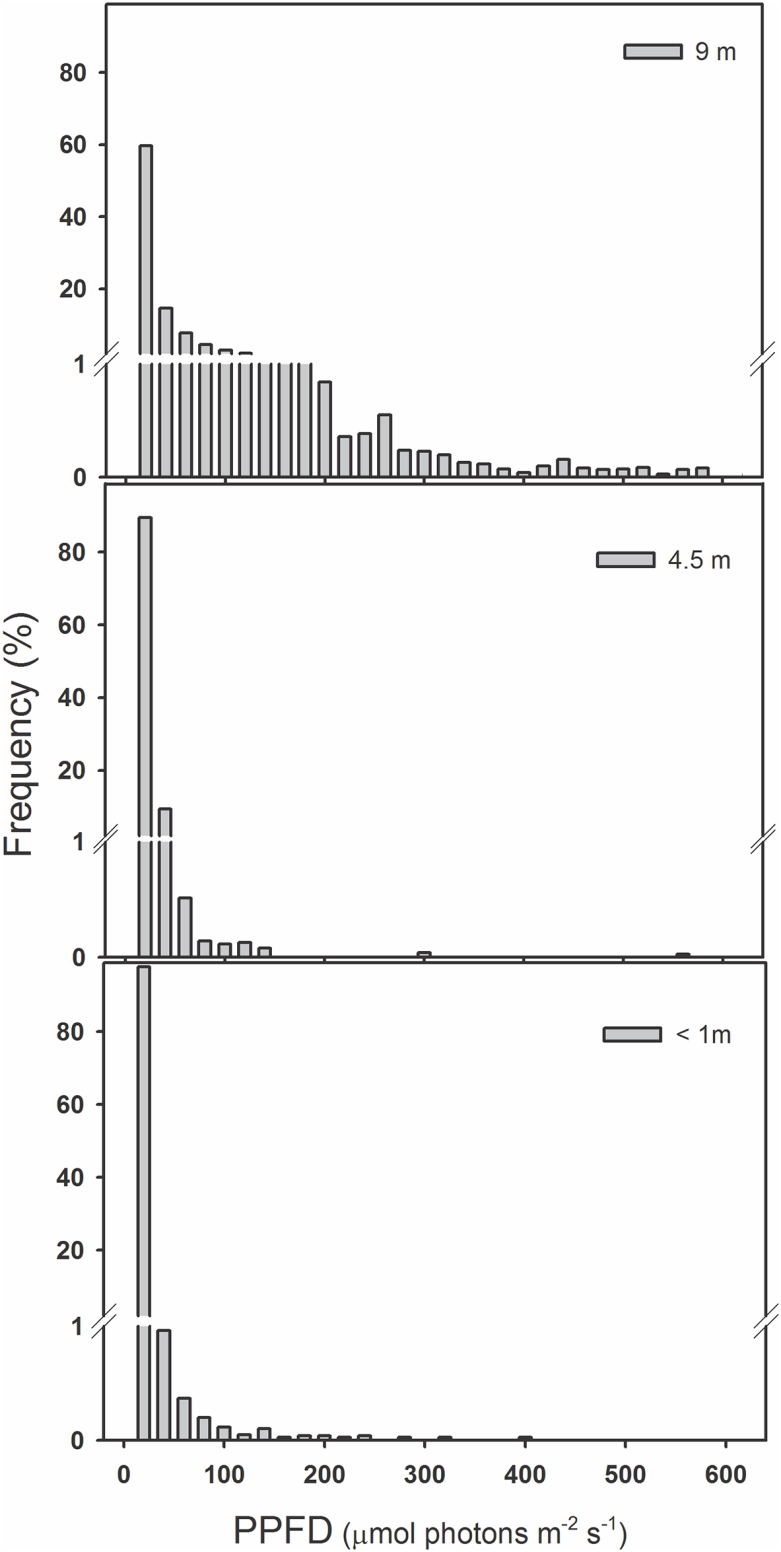
Light availability across a vertical gradient in the natural habitats of Hymenophyllaceae species in the Katalapi Park. Frequency of observed photosynthetic photon flux density (PPFD, μmol photons m^-2^ s^-1^) measured at three trunk heights: <1, 4.5 and 9 m using data collected by two different data-loggers from 01 October 2010 until 01 February 2011.

In a secondary forest of Southern Chile, the diversity and abundance of filmy ferns decrease with the host tree height [22]. While some filmy species are restricted to their hosts lower heights (i.e. <60 cm), where light availability is very low, (i.e. 10–100 μmol photons m^-2^ s^-1^), other species occupy the whole host height range, (reaching heights ≥10 m). Light availability at this height near the canopy, although unfrequently can exceeds 500 μmol photons m^-2^ s^-1^ for at least a couple of hours during the day in spring and summer (Fig 1 and S1 Table). This filmy ferns vertical distribution in the host tree is, at least to some degree, explained by the microhabitat characteristics, particularly the relative humidity and canopy openness [22].

The aim of this work was to study the photosynthetic light responses of two Hymenophyllaceae species and the relation of these with their contrasting distribution patterns in a secondary temperate rainforest of Southern Chile. For this purpose, we determined potential differences in light tolerance between *Hymenoglossum cruentum* (Cav) K. Presl. and *Hymenophyllum dentatum* (Cav.). *H*. *cruentum* is commonly found inhabiting the host lower heights (i.e. <60 cm) and/or most shaded places inside the forest, while *H*. *dentatum* is present along the whole host height range and/or in more light exposed sites. Therefore, we proposed that *H*. *dentatum* is more tolerant to high light intensities than *H*. *cruentum*, which is reflected in higher photosynthetic performance and better non-photochemical responses.

## Materials and Methods

### Study area and plant material

**Fig 2:**
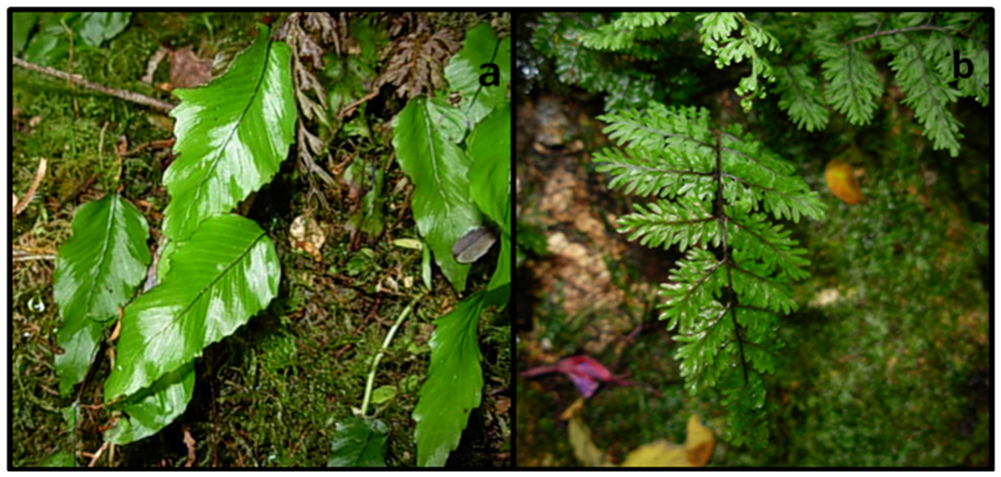
Two epiphytic Hymenophyllaceae species with contrasting vertical distribution in a secondary temperate rainforest (Katalapi Park, Puerto Montt, Chile). a) *Hymenoglossum cruentum*, and b) *Hymenophyllum dentatum*.

We compared light tolerance of two native epiphytic Hymenophyllaceae species: *Hymenoglossum cruentum* and *Hymenophyllum dentatum* (Fig 2). *H*. *cruentum* is an endemic Chilean species with plant height ranged 10–30 cm. Its fronds are entire and glabrous, with marginal sori placed on veins ends. In contrast, *H*. *dentatum* ranges 8–18 cm plant height. Its fronds are divided and have a dentate margin, hairy petiole, rachis and veins, and subaxilar sori [29].

Plants were collected at the Katalapi Park (41°31’12.0”S—72°45’02.3”W), located in the Cordillera de Quillaipe, near Puerto Montt, Chile. This area is characterized by a temperate evergreen interior forest, dominated by *Drimys winteri*, *Amomyrtus luma* and *A*. *meli*, *Laureliopsis philippiana*, *Nothofagus dombeyi* and *N*. *nitida*, *Raphythamnus spinosus*, *Weinmannia trichosperma*, and several Proteaceae species. It presents a temperate-humid climate with strong oceanic influence [30], with a mean air temperature of 15°C and 1900 mm of annual rainfall. This study was duly authorized by the owner and Park Director (Ms Ana María Vliegenthart), and it did not involve endangered or protected species.

Fern species were collected from different levels of the forest according to their vertical abundance. Individuals of *Hymenoglossum cruentum* were collected from parts of host <60 cm heights, while individuals of *Hymenophyllum dentatum* were collected between 60–200 cm host heights. For each species, plant material corresponded to bark sections that contained groups of adult filmy ferns, which were obtained from several host species. After collection in the field plant material was immediately transported to a nursery in the Laboratory of Plant Physiology at the Universidad de Concepción (Concepción, Chile). Filmy species were kept in the nursery during six days to recover them from stress produced by transport. Therefore, the time between plant material collection and their photosynthetic performance measurements was less than 1 week. In this period, both species were exposed to similar conditions to those found in the field. We controlled relative humidity (70–90%) by watering plants 2–5 times per day during 2 min, using a semi-automatic fog type irrigation system (own fabrication). Light intensity was maintained <70 μmol photons m^-2^ s^-1^, using a black mesh on all walls and roof to block part of incoming sunlight. All measurements were performed in detached fully hydrated fronds (placed in distilled water overnight).

### Vertical light gradient in the field

Photosynthetic photon flux densities (thereafter PPFD) were measured in the field to characterize vertical light gradients along trunk hosts (Table 1 and S1 Table). Two Quantum Smart sensors (S-LIA-M003, Hobo, Onset Computer Corporation, USA) were placed at three heights: <1m, 4.5 m and 9 m in trunks of two different stands of a well-grown secondary forest. Smart sensors were connected to an Energy logger (H22-001, Hobo Onset Computer Corporation, USA), and data were recorded every 15 min from October 01, 2010 to February 01, 2011 (i.e. Spring-summer period in the Southern Hemisphere).

**Table 1:** Vertical distribution of photosynthetic photon flux density (PPFD) in a secondary temperate rainforest of Southern Chile. Values correspond to daily mean ± SE (*n* = 2). Measurements were carried out in January 2011 in the Katalapi Park.

Trunk height (m)	Mean PPFD (μmol photons m^-2^ s^-1^)	Absolute maximum PPFD (μmol photons m^-2^ s^-1^)
< 1	32 ± 2	501
4.5	89 ± 4	548
9	623 ± 15	1,738

### Gas exchange measurements

For each species, photosynthetic light responses were measured on fully expanded but non-reproductive fronds (i.e. without sori), of a similar size and among those that already developed in the field. Hence, expected differences in light tolerance between species are attributed to contrasting microhabitats where fronds developed.

Six detached fronds from different individuals were used to obtain photosynthetic responses to different PPFDs. An Infrared Gas Analyzer (Portable photosynthesis measuring system, GFS-3000, WALZ, Effeltrich, Germany) was programmed to expose fronds to a progressive stepwise increase in PPFD. Photosynthetic response curves were built with seventeen PPFD levels from 0 to 300 μmol photons m^-2^ s^-1^. Each frond was exposed 3 min to each level of PPFD, to stabilize the net rate of CO_2_ Assimilation (A_n_) before each measurement. Therefore, the total time course for a whole light response curve was 51 min. A_n_ of each frond was measured at 15°C, 390 ppm CO_2_, and 95% relative humidity, to avoid suboptimal conditions produced by factors other than PPFD. This range of RH can be obtained in the GFS-3000 because it has and integrated H_2_O control via step motor for humidifying and drying with a range from 0 to nearly 100% RH preventing condensation (WALZ, Effeltrich, Germany). Before each measurement, fronds were hydrated for 24 h. A_n_ was fitted to a quadratic hyperbolic function using STATISTICA 7 Statsoft ^®^ software and following Lambers et al. (2008) [31]. From each curve, light compensation **(I**_**c**_ **)**, light saturation point **(I**_**s**_), and maximum rate of CO_2_ assimilation **(A**_**max**_ **)** were estimated. **I**_**c**_ corresponds to the PPFD where the rate of CO_2_ assimilation is balanced by the rate of CO_2_ production in respiration and photorespiration; **I**_**s**_ corresponds to the PPFD over which the rate of CO_2_ assimilation is maximal and insensitive to level of PPFD; and **A**_**max**_ is the light-saturated rate of gross CO_2_ assimilation (net rate of CO_2_ assimilation + dark respiration) at infinitely high irradiance [31]. Fronds were darkened during 30 min before the beginning of photosynthetic response curves to obtain the Dark Respiration rate **(R**_**d**_ **)**. In the case of *H*. *cruentum*, light steps used for fitting curves were limited to 50 μmol photons m^-2^ s^-1^ because above this PPFD, its net rate of CO_2_ assimilation abruptly dropped.

Given that *H*. *dentatum* fronds did not cover completely the area of the IRGA cuvette, we took a photograph of each frond inside the cuvette, and then we calculated its area by using Sigma scan^®^ software. With this information we corrected A_n_, A_max_, and R_d_ measurements as way as to standardize these parameters to an area of 4 cm^2^, which was the area of the IRGA cuvette used for gas exchange measurements in both fern species. No area corrections were made for *H*. *cruentum* because their fronds covered completely the area of the IRGA cuvette.

### Light energy partitioning at PSI and PSII

Simultaneous assessment of changes in P700 absorbance and PSII chlorophyll fluorescence were performed in four detached fronds of each filmy species using a Dual-PAM 100 measuring system (WALZ, Effeltrich, Germany). PPFD response curves were programmed using the scripting facility of the Dual-PAM 100 control software to expose each frond to successively increasing actinic light levels, with 3 min equilibration time at each level before measurements. These determinations were made at 15°C, kept constant by adding two metal collars around the Dual PAM-100 measuring heads and then connecting them to a cooling/heating circulator (Thermo Haake K15, Electron Corporation, Germany). Fully hydrated fronds were dark adapted during 30 min before each PPFD response curve. This dark adaptation is necessary to determine the intrinsic or maximal fluorescence of PSII (F_m_), which is used in NPQ calculations [3]. Photochemical quenching (qL) was calculated according to Kramer et al. 2004 [9].

We assessed the photochemical light responses of these filmy ferns through the following parameters: For PSII we calculated: **Y**_**PSII**_, photochemical quantum yield of PSII; **Y**_**NPQ**_, regulated heat dissipation quantum yield, and **Y**_**NO**_, non-regulated heat dissipation quantum yield. For PSI we calculated: **Y**_PSI_, photochemical quantum yield of PSI, **Y**_**ND**_, non-photochemical quantum yield caused by a donor side limitation, and **Y**_**NA**_, non-photochemical quantum yield caused by an acceptor side limitation [9, 32]. Recordings and calculations were performed with the Dual-PAM 1.7 data analyses and control software (WALZ, Effeltrich, Germany). Calculations of ETR_II_ (ETR_II_ = 0.5 x Abs x Y(II) x PPFD) and ETR_I_ (ETR_I_ = 0.5 x Abs x Y(I) x PPFD) given by the instrument were corrected using the actual average absorbance of fully hydrated fronds (n = 4) of each filmy fern species. For this, the absorbance (Abs) was calculated using the automatic routine for measurement PAR-absorptivity image of Imaging PAM-mini (Walz, Effeltrich, Germany). This measurement requires successive illumination of the samples with red (R) and NIR light and the capture of each remission image. The absorbance is calculated by the equipment software pixel by pixel as follows: Abs = 1- R/NIR. The average values for absorbance were 0.58 ± 0.03 for *H*. *cruentum* and 0.44 ± 0.07 for *H*. *dentatum* (mean ± SE, n = 4).

### NPQ components determination under photoinhibitory conditions

For each fern species, we determined fast and slow dark relaxing components of NPQ in five detached, adult, and fully expanded fronds of different individuals, which were previously dark adapted during 30 min to obtain the minimum and maximal fluorescence parameters (i.e. F_0_ and F_m_, respectively). The measurements were made with a FMS II modulated fluorimeter (Hansatech Instruments Ltd, UK), whose probe was connected to a LD2/3 camera (Hansatech Instruments Ltd, UK), to expose fronds to a LS2 white light source (Hansatech Instruments Ltd, UK). Measurements were made at 15°C, kept constant using a cooling/heating circulator (Thermo Haake K15, Electron Corporation, Germany), that was connected to LD2/3 camera. Fast (NPQ_f_) and slow (NPQ_s_) relaxing components were obtained essentially as described by Walters and Horton (1991) [33], analyzing the kinetics of Fm recovery after actinic light has been turned off, where NPQ_s_ = (Fm-Fm_r_)/Fm_r_, and NPQ_f_ = (Fm-Fm’)-(Fm-Fm_r_). Fm_r_ (value of Fm that would have been attained if only slowly relaxing quenching had been present) was obtained by extrapolation in a semi-logarithmic plot of maximum fluorescence yield versus time of data points recorded toward the end of the relaxation back to the time where the actinic light was removed. This graph was obtained after exposing 5 dark adapted fronds to a photoinhibitory treatment during 25 min, at an actinic light intensity of 1,500 μmol photons m^-2^ s^-1^, and 1h of recovery time in darkness.

### Pigments Analyses

Approximately 0.1 g fresh tissue fronds per fern species (n = 3) were collected from plants growing in the nursery. Fresh samples were immediately frozen in liquid nitrogen and stored at -80°C until analysis. Samples were powdered, and immediately a spatula tip of CaCO_3_ was added before extracting with 1 mL 100% HPLC-grade acetone at 4 ◦C under dim light. The extract was spun down and the supernatant was filtered through 0.45 μm syringe filter. Pigments, including chlorophyll *a* and *b*, β-carotene (β-car) and α-carotene (α-car), neoxanthin (Neo), and xanthophyll cycle pigments, violaxanthin (V), antheraxanthin (A) and zeaxanthin (Z) contents were measured by a high performance liquid chromatography (HPLC) method described by García-Plazaola and Becerril (1999) [34], with instrumentation and HPLC conditions modified by Sáez et al. (2013) [35]. De-epoxidation state (DEPS) of the xanthophyll pool was calculated as follow: DEPS = (V+0.5A)/(V+A+Z).

### Data analyses

Gas exchange parameters (i.e. R_d_, A_max_, Ic and Is) were compared between species using *t* tests. PPFD response curves were compared at two points, which corresponded to light saturation points (I_s_) of each species. At these points, differences between species for Y_PSII_, Y_NPQ_, Y_NO_, Y_PSI_, Y_ND_, Y_NA_, ETR_II_ and ETR_I_ were assessed using *t* tests. Differences between species for NPQ components, pigments contents and ratios were assessed using *t* tests as well. Alternative non parametric tests were used when assumptions of normality and homoscedasticity where not met after transformations [36].

## Results

### Gas exchange responses

**Fig 3:**
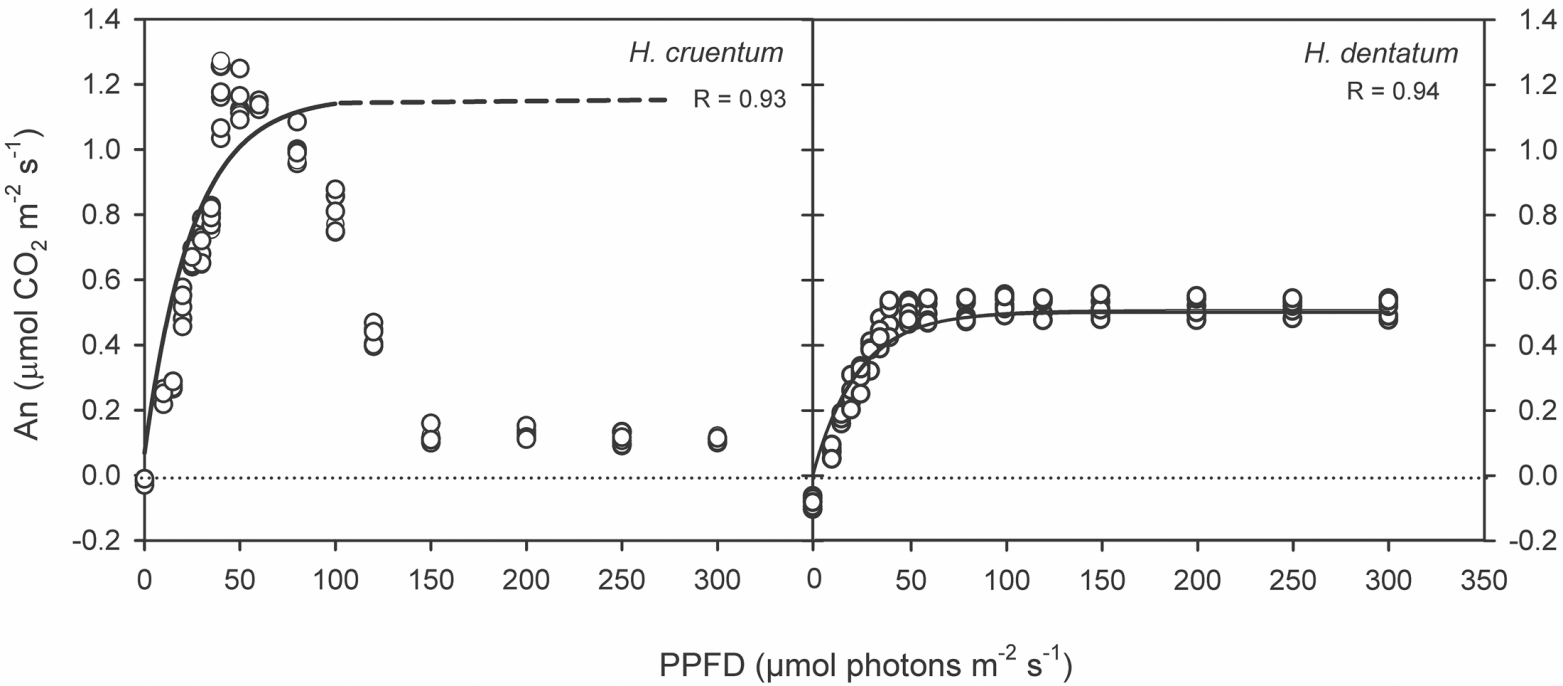
Dependency of CO_2_ net assimilation rate (A_n_, μmol m^-2^ s^-1^) on Photosynthetic Photon Flux Density (PPFD, μmol photons m^-2^ s^-1^) in two filmy fern species with contrasting vertical distribution: *Hymenoglossum cruentum* and *Hymenophyllum dentatum*. All replicates were plotted in the graph (*n* = 6). A black line indicates the fit curve for photosynthetic responses to light following Lambers et al. (2008) [31]. A dashed line indicates the portion of the fit curve where observed values for *H*. *cruentum* diverted from those expected values.

CO_2_ assimilation responses to an increase of photosynthetic photon flux density (PPFD) differed between species (Fig 3 and S2 Table). *Hymenophyllum dentatum* showed a mean A_max_ 2.2 times lower than *Hymenoglossum cruentum* (Table 2 and S3 Table; *t* = 30.1, *P* <0.0001).

**Table 2:** Photosynthetic performance of two filmy ferns with contrasting vertical distribution on tree trunks: *Hymenoglossum cruentum* and *Hymenophyllum dentatum*. Parameters were obtained from gas exchange measurements: R_d_, dark respiration; A_max_, maximum CO_2_ assimilation rate; I_c_, light compensation point; I_s_, light saturation point. Data are shown as mean values ± SE (*n* = 6).

	*H*. *cruentum*	*H*. *dentatum*
R_d_ (μmol CO_2_ m^-2^ s^-1^)	0.02 ± 0.01	0.08 ± 0.01***
A_max_ (μmol CO_2_ m^-2^ s^-1^)	1.16 ± 0.02	0.54 ± 0.01***
Ic (μmol photons m^-2^ s^-1^)	1.45 ± 0.07	4.9 ± 0.15***
Is (μmol photons m^-2^ s^-1^)	24.6 ± 0.07	40.5 ± 1.4**

Levels of significance:

**, *P* <0.001;

***, *P* <0.0001.

*H*. *dentatum* kept its A_n_ near A_max_ over a wide range of PPFDs. In contrast, *H*. *cruentum* declined its A_n_ with an increase in PPFD, especially above 60 μmol photons m^-2^ s^-1^, where A_n_ sharply decreased to less than 10% of A_max_ (Fig 3; S2 and S3 Tables). Both species showed low R_d_, with the lowest values recorded in fronds of *H*. *cruentum* (Table 2 and S3 Table; *t* = 9.5, *P* <0.0001). Light compensation point (Ic) was 3.2 times lower in fronds of *H*. *cruentum* than in fronds of *H*. *dentatum* (Table 2 and S3 Table; *t* = -21.2, *P* <0.0001). Similarly, light saturation point (Is) was 1.6 times lower in fronds of *H*. *cruentum* than in fronds of *H*. *dentatum* (Table 2 and S3 Table; *Z* = 2.9, *P* = 0.004).

### Light Energy partitioning

**Fig 4:**
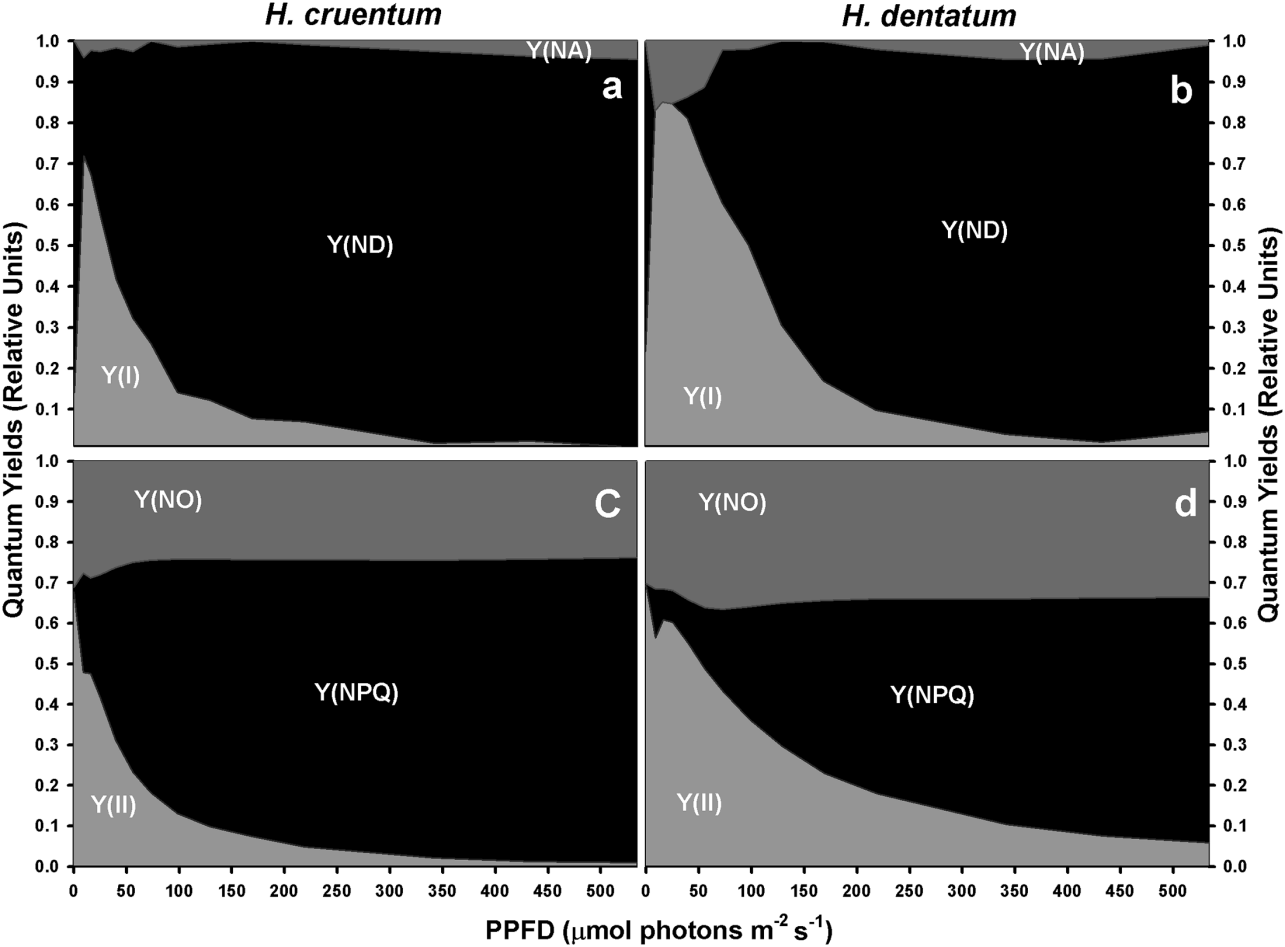
Changes in partitioning of absorbed excitation energy with increasing PPFD. This was measured at PSI (a, b) and PSII (c, d) level in fronds of *Hymenoglossum cruentum* and *Hymenophyllum dentatum*. A dashed line indicates the portion of the curve where comparisons between species were made. These points correspond to the light saturation points of *H*. *cruentum* at 24.6 μmol photons m^-2^ s^-1^ (Is_*H*.*cru*_) and of *H*. *dentatum* at 40.5 μmol photons m^-2^ s^-1^ (Is_*H*.*den*_), both obtained from gas exchange measurements. Values are shown as mean ± SE (*n* = 4).

Even when the light response pattern of PSI and PSII yields was very similar between the two ferns species, the compensation points of photochemical and non-photochemical processes, as well as the specific values at the corresponding I_s_, were different between filmy species (Fig 4 and S4 Table). First, *H*. *cruentum* yields compensated at irradiances below 50 μmol photons m^-2^ s^-1^; whereas those of *H*. *dentatum* did near to 100 μmol photons m^-2^ s^-1^.

Secondly, *H*. *cruentum* was photochemically less effective than *H*. *dentatum*. This was reflected by its 18% at Is_Hcru_ (*t* = 8.1; *P* <0.001) and 24% at Is_Hden_ (*t* = -5.9; *P* <0.01) lower Y_PSII_ (Table 3 and S4 Table), as well as by its earlier Y_PSII_ decrease to values below 0.1 at irradiances about 200 μmol photons m^-2^ s^-1^ less than *H*. *dentatum* (Fig 4c and 4d, S4 Table).

**Table 3:** Changes in PSII and PSI components in fronds of *Hymenoglossum cruentum* and *Hymenophyllum dentatum* measured at their respective light saturation points (Is). PSII parameters: Y_PSII_, photochemical quantum yield of PSII; Y_NPQ_, regulated heat dissipation quantum yield; Y_NO_, non-regulated heat dissipation quantum yield; and ETR_II_, electron transport at the PSII. PSI parameters: Y_PSI_, photochemical quantum yield of PSI; Y_ND_, non-photochemical quantum yield caused by a donor side limitation; Y_NA_, non-photochemical quantum yield caused by an acceptor side limitation, and ETR_I_, electron transport rate at the PSI. Values correspond to mean ± SE (*n* = 4).

Is	Is_H.cru_ = 24.6 (μmol photons m^-2^ s^-1^)		Is_H.den_ = 40.5 (μmol photons m^-2^ s^-1^)	
Species	*H*. *cruentum*	*H*. *dentatum*	*H*. *cruentum*	*H*. *dentatum*
**PSII**				
Y_PSII_	0.42 ± 0.01	0.60 ± 0.03**	0.31 ± 0.01	0.55 ± 0.04**
Y_NPQ_	0.30 ± 0.01	0.08 ± 0.02*	0.43 ± 0.02	0.11 ± 0.04**
Y_NO_	0.28 ± 0.01	0.32 ± 0.01*	0.26 ± 0.02	0.34 ± 0.01*
ETR_II_	2.98 ± 0.05	3.1 ± 0.20ns	3.64 ± 0.13	4.4 ± 0.44ns
**PSI**				
Y_PSI_	0.62 ± 0.04	0.85 ± 0.10^ns^	0.44 ± 0.04	0.60 ± 0.14*
Y_ND_	0.40 ± 0.05	0.00 ± 0.00*	0.57 ± 0.05	0.06 ± 0.04**
Y_NA_	0.03 ± 0.03	0.15 ± 0.10^ns^	0.02 ± 0.01	0.14 ± 0.04*
ETR_I_	4.44 ± 0.23	3.51 ± 0.79^ns^	5.24 ± 0.36	5.56 ± 1.39^ns^

Levels of significance: ns, not significant;

*, P <0.05;

**, P <0.001.

**Fig 5:**
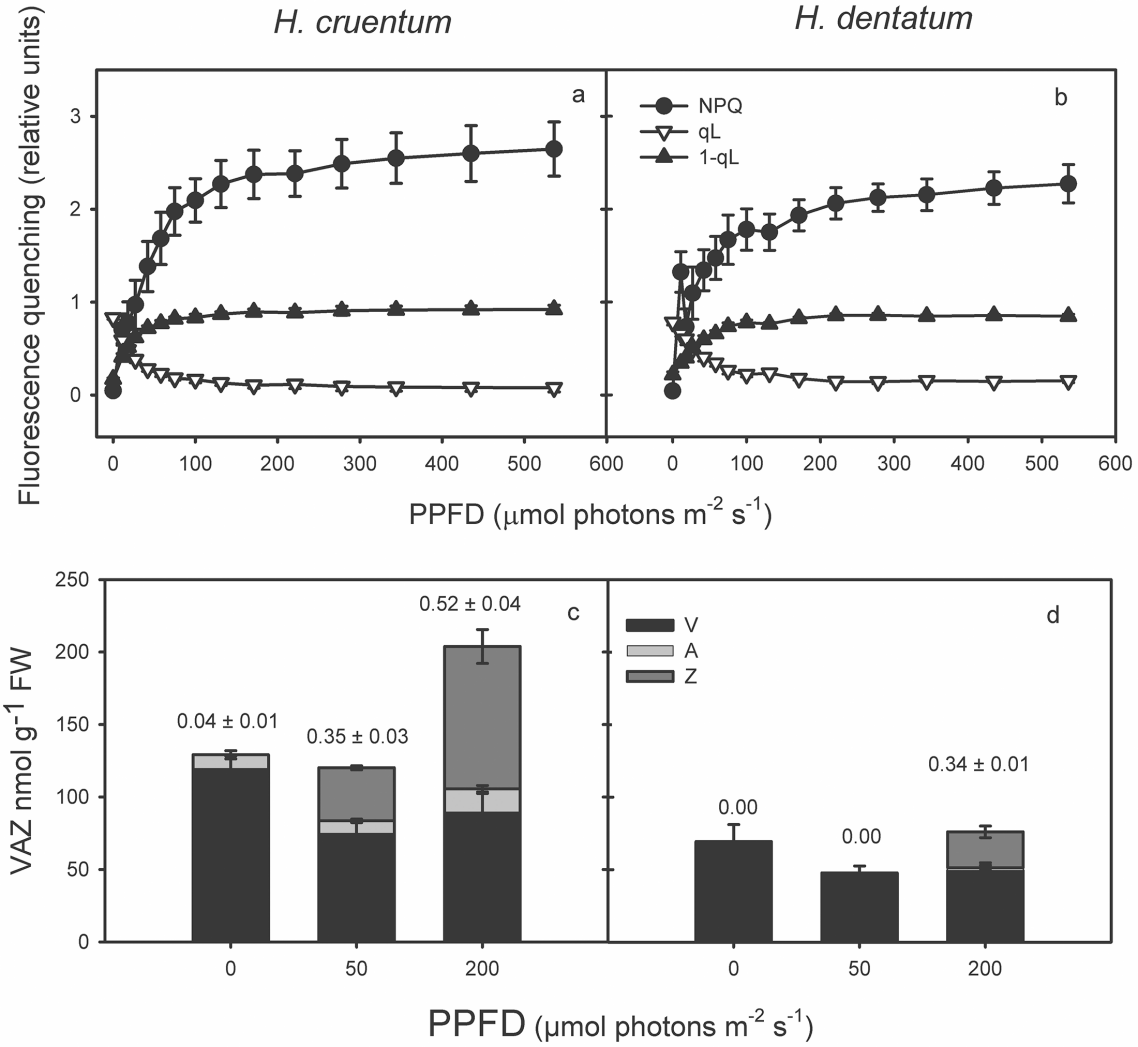
Light response curves of fluorescence derived quenching parameters NPQ, qL, and 1-qL, expressing non-photochemical quenching, the proportion of PSII open reaction centers (P680-Qa+) and the redox state of Qa, the primary acceptors of PSII for *H*. *cruentum* (a) and *H*. *dentatum* (b), respectively. Values correspond to the average of *n* = 3 ± SE. The xanthophyll cycle pool pigments and de-epoxidation state at three light points 0, 50 and 200 μmol photons m^-2^s^-1^ are shown for *H*. *cruentum* (c) and *H*. *dentatum* (d). Pigment determination was performed by HPLC. Values correspond to the average *n* = 3 ± SE, the numbers on top of each bar correspond to DEPS.

Such results coincide with the slightly lower proportion of open reaction centers of PSII (qL) in *H*. *cruentum* at saturating light intensities (Fig 5a and 5b, S5 Table). Hence, the redox state of the primary acceptor of PSII Qa-pool, estimated as 1-qL, was slightly higher in *H*. *cruentum* than in *H*. *dentatum*, being also saturated at lower PPFD.

The discrepancies in the ability to use the absorbed light energy were consistent with the corresponding proportion of energy dissipated as heat. Specifically, *H*. *cruentum* was the species with higher Y_NPQ_ across the entire experimental light curve (Fig 4c and 4d). The maximum differences between the species were found at both saturating light intensities, being the Y_NPQ_ values of *H*. *cruentum* a 22% at Is_Hcru_ (*Z* = 2.3; *P* <0.05) and a 32% at Is_Hden_ (*t* = 7.1; *P* <0.001) higher than *H*. *dentatum* (Table 3). Besides its general higher thermal dissipation at PSII level, *H*. *cruentum* also exhibited higher PSI non-photochemical quenching (Y_ND_ + Y_NA_) at the lower irradiances (Fig 4). In spite of such concomitance, a striking result was the different limitations that cause PSI non-photochemical quenching in each filmy fern at light intensities below 70 μmol photons m^-2^s^-1^. In *H*. *cruentum*, the PSI thermal dissipation was always caused by a donor side limitation (Fig 4a). However, in *H*. *dentatum* such dissipation was mostly conditioned by an acceptor side limitation (Fig 4b). In fact, at Is_Hden_, the *H*. *cruentum* Y_ND_ was a 51% higher than Y_ND_ in *H*. *dentatum* (*t* = 7.7; *P* <0.001); while the *H*. *dentatum* Y_NA_ was a 12% higher than *H*. *cruentum* (*t* = -2.8; *P* <0.05). The latter and coincident interspecific donor side limitation is consistent with the corresponding light-induced decreases in Y_PSII_ (Fig 4). Therefore, at any given PPFD, the excitation pressure of PSII and thermal energy dissipation tend to be higher for *H*. *cruentum* than *H*. *dentatum* (Fig 5a and 5b, S5 Table).

**Fig 6:**
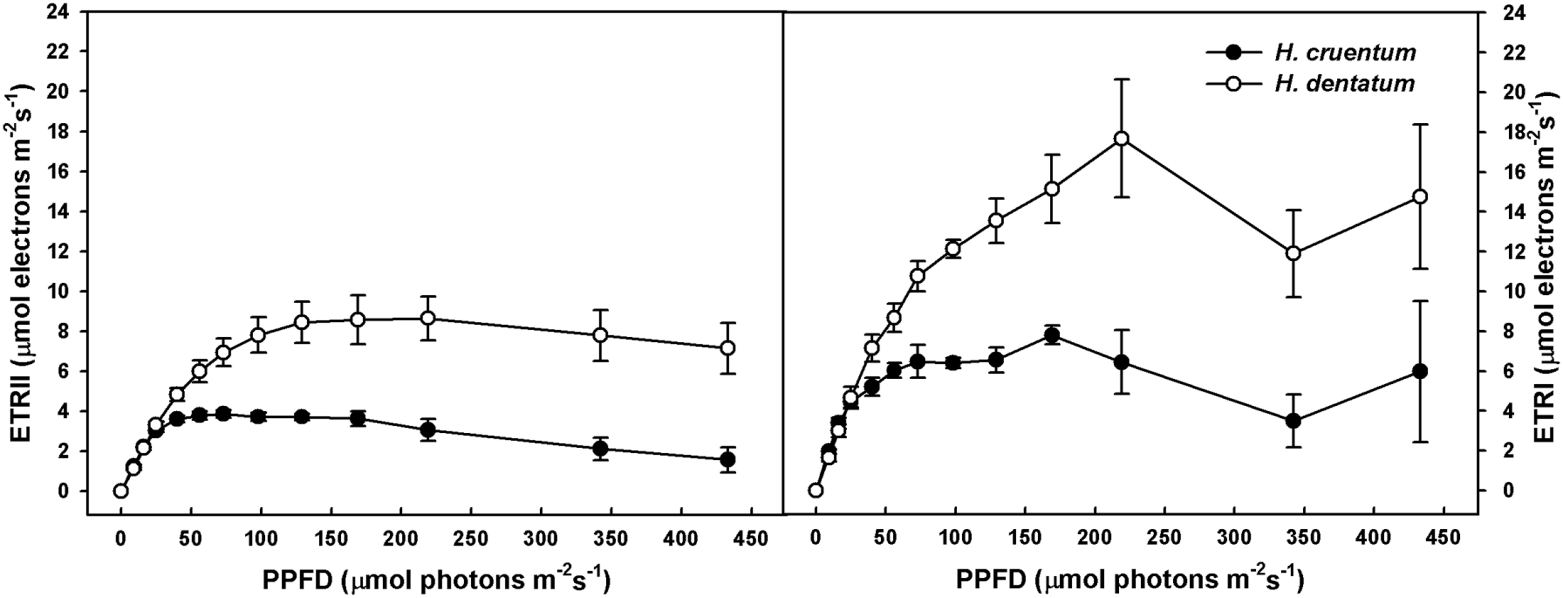
Light response curves of PSII (ETR_II_) and PSI (ETR_I_) electron transport rates in fronds of *H*. *cruentum* and *H*. *dentatum*. Detached fronds were fully hydrated overnight and then dark adapted during 30 min. PPFD response curves were programmed using the scripting facility of the Dual-PAM 100 control software. Each frond was exposed to successively increasing actinic light levels (0 to 436 μmol photons m^-2^ s^-1^), with 3 min equilibration time at each light level before the application of saturating pulses. Values correspond to the mean ± SE (*n* = 4).

Concerning Y_NO_ values, these were slightly different between the two species across all the irradiances applied. Actually, at both saturating light intensities, *H*. *dentatum* exhibited a 4% at Is_Hcru_ (*t* = -2.7; *P* <0.05) and an 8% at Is_Hden_ (*t* = -3.4; *P* <0.05) higher values than *H*. *cruentum* (Table 3). Finally, and regarding ETRs, there were not interspecific differences in both ETR_I_ and ETR_II_ within the light saturation range for net photosynthesis of both species (from 24.6 to 40.5 μmol photons m^-2^ s^-1^, Table 3). However, above 50 μmol photons m^-2^ s^-1^ *H*. *dentatum* exhibited about 100% higher ETR_I_ and ETR_II_ than *H*. *cruentum* (Fig 6).

In both species ETR_II_ were saturated at higher PPFD than net photosynthesis. This is a distinctive interspecific feature, where the ETR_II_ of *H*. *dentatum* saturated at PPFDs above 100 μmol photons m^-2^ s^-1^ while the ETR_II_ of *H*. *cruentum* saturated at about 50 μmol photons m^-2^ s^-1^. After saturation, ETR_II_ tended to decrease in both species being remarkable in *H*. *cruentum*, reaching at the last light step about 50% of its maximal ETR_II_. In the case of ETR_I_, saturation was not clear in *H*. *dentatum*, which exhibited a sustained increase until near 225 μmol photons m^-2^ s^-1^ (Fig 6). ETR_I_ in *H*. *cruentum* was saturated at about 50 μmol photons m^-2^ s^-1^ (Fig 6).

### NPQ components under photoinhibition

Photoinhibition at 1,500 μmol photons m^-2^ s^-1^ for 1h rendered no differences for the total NPQ between filmy fern species (Table 4; *t* = 1.9, *P* = 0.097), neither in the separated comparisons of fast (*t* = 2, *P* = 0.079) nor in the slow NPQ components (*t* = 0.9, *P* = 0.405). Most of the NPQ measured in fronds of these species corresponded to the fast component (NPQ_f_), being 84 and 91% of the total NPQ in *H*. *dentatum* and *H*. *cruentum*, respectively (Table 4 and S6 Table).

**Table 4:** Components of NPQ measured in fronds of *Hymenoglossum cruentum* and *Hymenophyllum dentatum* under photoinhibitory conditions. NPQ_f_ corresponds to the fast dark relaxing component and NPQ_s_ corresponds to slow dark relaxing component. Values are shown as mean ± SE (*n* = 5).

	*H*. *cruentum*	*H*. *dentatum*
NPQ	3.59 ± 0.54	2.42 ± 0.32^ns^
NPQ_f_	3.25 ± 0.52	2.03 ± 0.312^ns^
NPQ_s_	0.34 ± 0.06	0.39 ± 0.03^ns^

Levels of significance were ns, not significant.

### Pigments contents

There were significant differences in Chlorophyll contents between filmy species (Table 5 and S6 Table). Chlorophyll *a* and *b* contents were higher in fronds of *H*. *cruentum* than in *H*. *dentatum* (*t* = 3.1, *P* = 0.038). However, Chl *a*:*b* ratio was similar in fronds of *H*. *dentatum* and in *H*. *cruentum* (*t* = -1.7, *P* = 0.165). Neoxantin (Neo), a xanthophyll associated to the light harvesting complex II (LHCII), was 86% higher in *H*. *cruentum* than *H*. *dentatum (t* = 3.2, *P* = 0.034). The content of β-carotene (β-car), a pigment preferentially associated to the core of reaction centers of both photosystems, showed non-significant differences between species (*t* = 0.3, *P* = 0.790). The higher ratio Neo/β-car exhibited by *H*. *cruentum* (Table 5 and S6 Table; *t* = 12.4, *P* = 0.0002) suggests a bigger proportion LHC/RC in this species than in *H*. *dentatum*. In addition, *H*. *cruentum* exhibited much higher contents of α-carotene than *H*. *dentatum (t* = 6.8, *P* = 0.002).

**Table 5:** Pigments content and their ratios of two Hymenophyllaceae with contrasting vertical distribution on the host tree trunk in a temperate rainforest of Southern Chile. Values are shown as mean ± SE (*n* = 3).

	*H*. *cruentum*	*H*. *dentatum*
Chlorophyll *a* (nmol g^-1^ FW)	2,541 ± 241	1,403 ± 278*
Chlorophyll *b* (nmol g^-1^ FW)	1014 ± 101	541 ± 123*
Total chlorophyll (nmol g^-1^ FW)	3,555 ± 343	1,943 ± 401*
Chlorophyll *a*:*b* ratio	2.51 ± 0.01	2.63 ± 0.07^ns^
α-carotene (nmol g^-1^ FW)	115 ± 16	6 ± 2*
β-carotene (nmol g^-1^ FW)	96 ± 10	90 ± 21^ns^
Neoxanthin (nmol g^-1^ FW)	185 ± 15	99 ± 23*
Neo/β-car	1.94 ± 0.04	1.11 ± 0.06**

Levels of significance: ns, not significant;

*, *P* <0.05;

**, *P* <0.001.

## Discussion

Our results confirm earlier findings which have characterized Hymenophyllaceae species as shade plants [23–26]. This was reflected by low light-saturated rates of net photosynthesis and low light compensation points in *Hymenoglossum cruentum* and *Hymenophyllum dentatum*. Although both fern species are adapted to low light intensities, *H*. *dentatum* has somehow higher light requirements than *H*. *cruentum*. This suggests that contrasting vertical distribution of both filmy species is related, at least in part, to differences in their light tolerance. Specifically, *H*. *dentatum* showed A_max_ values lower than *H*. *cruentum* (Table 2); however, it was able to maintain its An maximum value across a wide range of light intensities (Fig 3). Contrasting to this, the higher A_n_ values of *H*. *cruentum* declined abruptly after 60 μmol photons m^-2^ s^-1^ (Fig 3). Such difference between the photosynthetic behaviors of these filmy ferns is not fully understood, but we could speculate two nonexclusive explanations. First, it could be attributed to an earlier and more intense dynamic photoinhibition of *H*. *cruentum* at irradiances that exceed its optimum activity (24.6 μmol photons m^-2^ s^-1^). This is supported by the concomitant sharp increase in the fraction of energy dissipated as heat (Y_NPQ_), which overpassed Y_PSII_ at 50 μmol photons m^-2^s^-1^, and also by the highest saturated NPQ observed in *H*. *cruentum* (Fig 5c and 5d). The idea that a chronic photo-inhibition is not likely comes from a photoinhibition assay. In this, the dark relaxation kinetics of NPQ was measured in *H*. *cruentum* and *H*. *dentatum* exhibiting about 80% and 90% of NPQ recovered within the fast relaxation kinetic component (NPQ_f_), respectively. Therefore, both species were able to safely dissipate the excess absorbed energy, probably through xanthophyll dependent heat dissipation (Fig 5). The second possible explanation is that A_max_ was reduced because an increased CO_2_ diffusion limitation. Despite these species lack stomata, preliminary measurements and anatomy-based estimates suggest that Hymenophyllaceae do have a strong diffusional limitation to photosynthesis, which is associated to their lack of true mesophyll, presence of very thick cell walls and few chloroplasts with a disperse distribution inside cells (Jaume Flexas pers. comm.). The hydration level in poikilohydrous photosynthetic tissues may influences diffusion of CO_2_ [37]. In our case, measurements were done at the fully hydrated state. The light response curve took 51 minutes at 95% RH and 15°C inside the IRGA cuvette. This determined a VPD about 0.085 KPa, an order of magnitude lower than VPD experienced by plants in the field [38]. Therefore, the chance of dehydration during measurements is low, especially in *H*. *cruentum* that dehydrates at half of the dehydration rates of *H*. *dentatum* [39]. The high water status of the fronds in our experiment may have reduced CO_2_ diffusion since liquid phase diffusion of CO_2_ is four orders of magnitude lower than in gas phase [40]. It is likely that levels of internal CO_2_ of *H*. *cruentum* were enough to sustain low rates of photosynthesis at low light at the beginning of the measurement, but as the rate of CO_2_ assimilation increased with PPFD, internal CO_2_ concentration could have dropped to a limiting level, causing the abrupt drop of net photosynthesis. This hypothetical explanation concerning CO_2_ diffusion limitation has however, a contradictory result in our analyses of PSI yields. If CO_2_ limitations have caused this drop in A_n_, then we would have expected a high PSI acceptor side limitation in *H*. *cruentum*. Nevertheless this was not the case (Table 3). A possible explanation to such contradictory result is that *H*. *cruentum* is able to use an alternative to NADP^+^ electron sink at high irradiance maintaining a high ETR_II_/net photosynthesis ratio. *H*. *cruentum* exhibited a sustained decrease of ETR_II_ after reaching only 1.5 μmol electrons m^-2^ s^-1^ at 430 μmol photons m^-2^ s^-1^, increasing the ETR_II_/net photosynthesis ratio from about 4 to 15 ê/CO_2_. Considering this, it is likely that alternative electron sinks are also operative for *H*. *dentatum* which exhibited much higher ETR_II_/net photosynthesis ratio than *H*. *cruentum* (17 ê/CO_2_). The use of alternative electron sinks is a common physiological response in poikilohydric bryophytes [41]. Preliminary measurements of ETR under low oxygen (2%) near 150 μmol photons m^-2^ s^-1^ have shown a reduction of 30% of maximal relative ETR of *H*. *cruentum* (unpubl. data). This suggests oxygen as an electron alternative sink. Experiments to demonstrate the presence of alternative electron sinks in these two filmy ferns and their capacity to deal with the resulting reactive oxygen species are under way.

On the other side, low light compensation and saturation points of both filmy ferns (i.e. <50 μmol photons m^-2^ s^-1^) were consistent with those reported for *Hymenophyllum tunbridgense* and *H*. *wilsonii*, two British filmy species with contrasting distribution. According to the author, *H*. *tunbridgense* exhibited the lowest light compensation point, which coincides with its localized and sheltered distribution [42]. In a broad sense, differences in photosynthetic performance showed by *H*. *dentatum* and *H*. *cruentum* are characteristic of adaptation to sun and shade habitats [10], and were consistent with PPFD differences obtained in their respective habitats. Specifically, *H*. *dentatum* is able to tolerate moderate light intensities or even sunflecks (about 1,500 μmol photons m^-2^ s^-1^) at the upper parts of the host, while *H*. *cruentum* is restricted to shade sites in the forest (PPFD <100 μmol photons m^-2^ s^-1^; see Table 1 and Fig 1).

As was mentioned above, differences in light responses curves between *H*. *cruentum* and *H*. *dentatum* were consistent with the results found in photosynthetic performance (Fig 3). For instance, both filmy species were more efficient in dissipating the absorbed energy by photochemical reactions (Y_PSII_) at their respective light saturation points (Table 3), but at higher irradiances, they change their strategy of photo-protection to heat dissipation (Y_NPQ_) (Fig 4a and 4b). Regarding to PSI, the different initial pattern of donor and acceptor side limitations in both filmy ferns (Fig 4a and 4b), could be attributed to differences in their respective rubisco carboxylation speeds and/or rubisco contents. If this were true, it can be assume that *H*. *cruentum* would be the species that fulfills such physiological characteristics, having higher A_max_ at very low light intensities but lower A_n_ at higher irradiances (Fig 3). In the same way, the concomitant Y_ND_ increase and Y_NA_ decrease in *H*. *dentatum* at irradiances that exceeds its photosynthetic saturating points, as well as its higher and longer maintained Y_PSI_ across a wide range of light intensities, suggests a possible participation of PSI cyclic electron flow in the maintenance of *H*. *dentatum* A_n_ during its exposure to permanent high or increasing light intensities (Fig 3b). Further studies should be conducted in order to understand the importance of PSI activity in these species adapted to light limiting conditions.

NPQ is primarily a measure of non-radiative dissipation of excitation energy and may be seen as essentially photo-protective path for higher plants and especially for poikilohydrous bryophytes and ferns [10, 39, 41]. Contrary to differences observed between species in Y_NPQ_ around their respective light saturation points, similar levels of total heat dissipation (NPQ) were observed under photoinhibitory conditions. Interestingly, most of NPQ recorded in fronds of both species corresponded to the fast NPQ component, which is involved in protection of PSII against over-excitation, indicating that although *H*. *cruentum* and *H*. *dentatum* are considered shade plants, they are able to manage excess of light energy absorbed, thus decreasing their probabilities to suffer photo-damage.

Chlorophyll contents are in line with the contrasting vertical distribution of filmy species (Table 5). *H*. *cruentum*, which inhabits the shadiest strata showed a higher chlorophyll *a* and *b* contents than *H*. *dentatum*, which could enable it maximizes the capture of limiting photons under low-light conditions [15, 25]. However, Chlorophyll *a*:*b* ratio in fronds of *H*. *cruentum* and in *H*. *dentatum* were similar. Both species showed Chl *a*:*b* ratio around 2.5, which is concordant with previously reported values for these species [22], and similar to those reported for shade Malayan ferns species [43]. A low Chl *a*:*b* ratio is generally indicative of a plants possessing a large proportion of Chl *a/b*-binding light-harvesting-complexes respect to reaction centers, an adaptation that is typical of plants adapted to low light environments [25, 44]. A high proportion Chl *a/b*-binding light-harvesting-complexes respect to reaction centers is also supported by a low Neo/β-car ratio observed in *H*. *cruentum* (Table 5). Neoxanthin is present in the LHCII while β-carotene is mainly in the reaction center core of both photosystems [45, 46]. According to the literature, higher plants do not present differences in Neo contents on chlorophyll basis. This is also the case in our filmy ferns; both have about 50 mmol per mol of total chlorophyll. Similar levels of β-carotene have been observed in shade leaves of angiosperms [47]. Interestingly, *H*. *cruentum* exhibited a significant higher content of α-carotene, which coincides with reported levels in deep shade species [48]. Nonetheless, β-carotene contents were not significantly different between both filmy species. Furthermore, *H*. *cruentum* had the highest xanthophyll cycle pigment pool and de-epoxidation level (Fig 5c and 5d). In general, this pattern is somehow different from angiosperms; because shade plants, usually exhibit lower β-carotene, lower xanthophyll cycle pigments pool and lower de-epoxidation state than sun plants [47, 48].

Ecophysiological studies with Hymenophyllaceae species are valuable empirical evidence of an evolutionary shift of adaptive strategy from typical vascular plant adaptation to the poikilohydry most typical of bryophytes [49]. Filmy ferns have unistratose leaves and are likely to be CO_2_ diffusion-limited at high irradiance [50]. In the case of bryophytes, sun-adapted species that suffer desiccation intermittently can maintain high rates of photosynthetic electron transport using oxygen as the only electron sink. This photoreduction of oxygen is linked with development of the high NPQ and associated photoprotection [51]. In this context, our results particularly invite considerations from two points of view: How do they highlight the different behavior of two filmies in the field, and what do they tell us about the adaptation and broad ecological niche of Hymenophyllaceae in general? For fundamental physical reasons, small ecto-hydric plants with monolayer leaves, in which external capillary water plays a physiologically essential role, are inherently best adapted to function at rather low light conditions [52, 53]. In our study, fronds of filmy ferns were always measured at full hydrated state, but in the field their vertical distribution also implies habitat differences explained by relative humidity [22]. Thus, it should be essential to assess light tolerance differences under wet conditions that represent this natural scenario. Thereby, we could know if photosynthetic performance and photoprotection responses observed in *H*. *cruentum* and *H*. *dentatum* underlie physiological responses against combined stresses of bright light and repeated cycles of drying and rewetting, especially at the upper strata of the forest.

## Conclusions

Our photosynthetic characterization under full hydration state confirms that *Hymenoglossum cruentum* and *Hymenophyllum dentatum* are shade plants. However, their differences in photosynthetic performance and photoprotection responses suggest that they possess different levels of light tolerance, having *H*. *dentatum* more plasticity for using a wider range of light. This is consistent with the contrasting vertical distribution of these filmy ferns previously observed in the rainforest of Southern Chile.

## Supporting Information

S1 Table Light availability in the Katalapi Park (Puerto Montt, Chile). (XLSX)

S2 Table Gas exchange measured in two Chilean filmy ferns. (XLSX)

S3 Table Photosynthetic performance parameters obtained from gas exchange measurements. (XLSX)

S4 Table Light energy partitioning at PSII and PSI. (XLSX)

S5 Table Quenching parameters derived from light response curves of Chlorophyll fluorescence. (XLSX)

S6 Table Pigment contents in fronds of *H*. *cruentum* and *H*. *dentatum*. (XLSX)
